# A low-cost and open-source mini benchtop centrifuge for molecular biology labs

**DOI:** 10.1016/j.ohx.2022.e00328

**Published:** 2022-06-16

**Authors:** Viktor Soma Poór

**Affiliations:** University of Pécs, Medical School, Department of Forensic Medicine, Hungary

**Keywords:** Centrifuge, Molecular biology, Labware

## Abstract

The aim of this manuscript is to provide all the necessary information to build a simple benchtop mini centrifuge that is able to remove droplets and liquid film from the inner wall of PCR tubes and PCR strips following vortexing. Users can choose between two rotors: one can hold up to six tubes (1.5/2 ml, or 500 µl and 200 µl tubes with adapters) or two PCR strips.

The design consists of only three printed parts (optimized to be printed without supports) and cheap off-the-shelf components (80 mm computer fan, switches, food container and power supply). It can be assembled with basic soldering skills, no programming needed.

While the total cost of materials is only 20 €, when compared to its commercial counterparts, this DIY benchtop spinner was equally capable to remove droplets. Comparing noise levels this model was quieter than an Eppendorf 5424, BioSan MSC-6000 or Kisker biotech centrifuges.

Because it is designed to perform a very generic and common task, it could be useful for a very wide array of laboratories and researchers: molecular biology, genetics, microbiology, student labs, biohacking labs, etc.

## Graphical abstract

**Figure:**
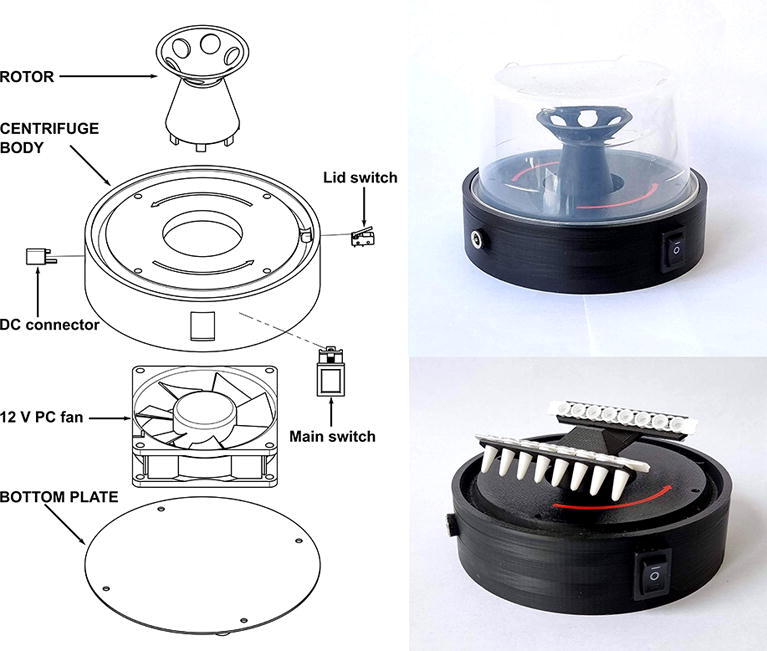

**Specifications table:** 

Hardware name	Open-source mini benchtop centrifuge
Subject area	• Biological sciences (e.g., microbiology and biochemistry)
Hardware type	• Biological sample handling and preparation
Closest commercial analog	Fisherbrand™ Standard Mini-Centrifuge (Thermo Fisher Scientific)
Open source license	Creative Commons Attribution-ShareAlike 4.0 International License
Cost of hardware	20 EUR
Source file repository	https://doi.org/10.17632/sy2vrhj227.1 Mendeley Data: https://data.mendeley.com/datasets/sy2vrhj227/1

## 1 Hardware in context

Small benchtop centrifuges are ubiquitous in molecular biology, genetics of microbiology labs. Often they are only used to remove liquid film or droplets from the inner wall of plastic tubes after homogenization. Mixing of reagents by vortexing is a crucial and often needed step in several methods in molecular biology or genetics. One prime example is quantitative real-time PCR, where master mixes are often used. Thin liquid layer film adhering to the inner surfaces of centrifuge tubes is a major contributor to ‘pipetting losses’. A simple ‘spin-down’ centrifugation can minimize these costly losses while it does not require precise RPM control or a particularly high speed, a simple centrifuge would suffice to remove droplets from the inside wall of plasticware. Also, quantitative real-time PCR applications tend to use PCR strip tubes, where eight small, 200 µl tubes are connected. While removing droplets of reagents from the caps of these tubes significantly improves the reliability of real-time PCR, it is relatively hard to come by small centrifuges which can be loaded with PCR strips.

**Fig. 1:**
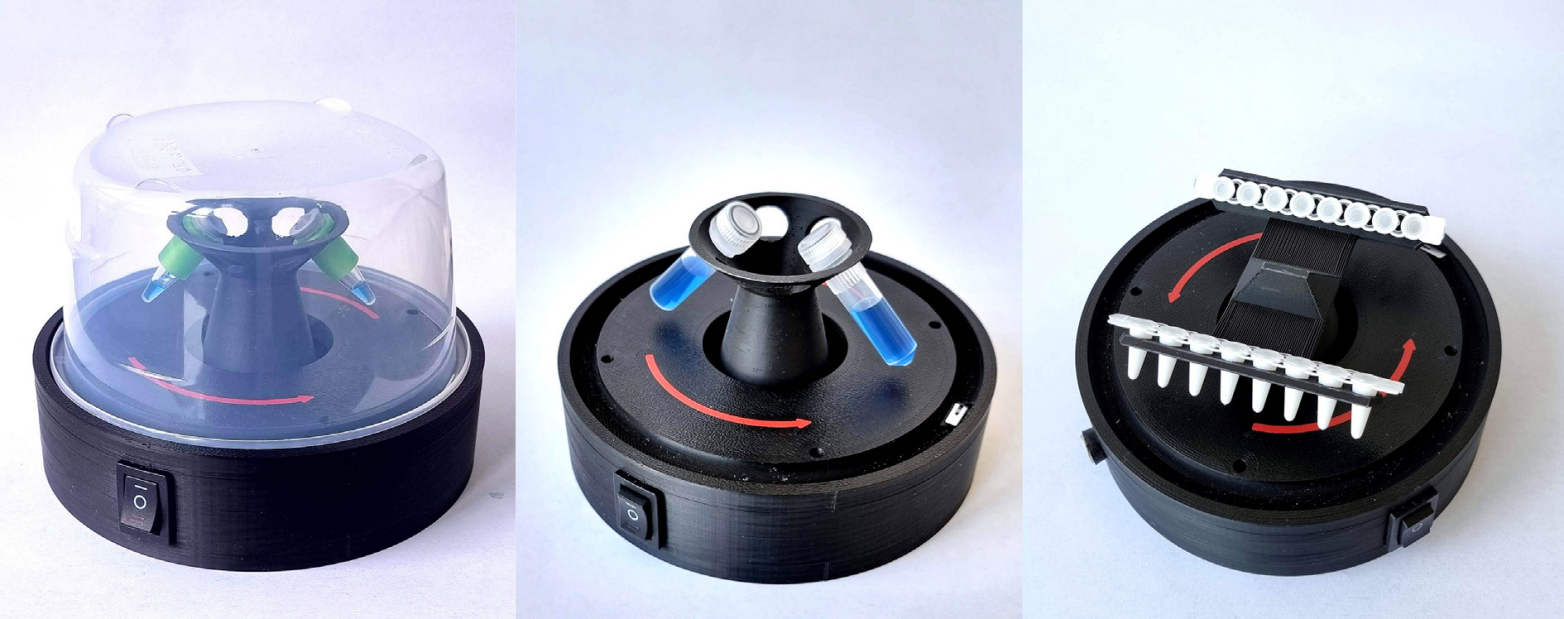
Illustration of the assembled mini centrifuge with a six-tube rotor and lid (left), six tubes rotor (middle) and with a PCR strip rotor (right). The six tube rotor holds plastic tubes of the most common sizes: 1.5/2 ml, 500 µl and 200 µl with the appropriate adapter. The PCR strip rotor can hold full length and short strips.

To decrease the risk of cross-contamination, it is advised that DNA extraction, and pre and post-PCR areas are physically separated. All these workspaces could be equipped with these affordable small centrifuges to spin down the reagent tubes (See Fig. 1).

## 2 Hardware description

The main features of the mini centrifuge:

• Simple design with only three printed parts and generic, affordable off the shelf components.
• It is able to spin six tubes or two PCR strips simultaneously.
• It has the features, “look and feel” of a commercial mini centrifuge.
• Minimal footprint, to maximize benchtop space.
• The motor only spins if the lid is locked on top.
• All parts can be printed with small, entry-level 3D printers. No supports are necessary.
• Basic soldering skills should suffice. No programming is needed.

Other projects already produced several surprisingly different laboratory centrifuge designs. One end of the simplicity spectrum is the paper [1] or 3D printed [2] hand driven designs. The DremelFuge [3] model showed that rotary tools can power high-speed centrifuges. These instruments spin tubes without any lids, which can increase the chance of contamination in the lab. The OpenFuge [4] and DIYbio Centrifuge [5] projects use specific motors and drivers to control them, which might be harder to source. OpenFuge also uses laser-cut panels, nowadays 3D printed parts are much more affordable. Probably the most similar to the device described in this manuscript is the open source centrifuge published by Warejoncas et al. [6] but it can spin only two tubes at the same time. The fan is placed into a container box, which should be opened and closed with two hands. Also, the authors chose to remove the blades of the fan, which can result in an imbalance. Bhupathi describes Mobilfuge, which uses 3D printed parts with an USB powered fan [7]. Advantages that it does not need soldering and can reach high RPM, but it is able to spin only two tubes, and also lacks safety lid switch. None of these centrifuges listed above are able to spin PCR strip tubes.

***Design files***

## 3 Design files summary

All the design files are available at https://doi.org/10.17632/sy2vrhj227.1 (Mendeley Data: https://data.mendeley.com/datasets/sy2vrhj227/1).

**Table:** 

**Design file name**	**File type**	**Open source license**
Centrifuge body	STL and STEP	Creative Commons Attribution-ShareAlike 4.0 International License
Centrifuge body – compatibility	STL and STEP	Creative Commons Attribution-ShareAlike 4.0 International License
Arrows	STL and STEP	Creative Commons Attribution-ShareAlike 4.0 International License
Bottom plate	STL and STEP	Creative Commons Attribution-ShareAlike 4.0 International License
Rotor – 32.5 mm	STL and STEP	Creative Commons Attribution-ShareAlike 4.0 International License
Rotor – PCR strip – 32.5 mm	STL and STEP	Creative Commons Attribution-ShareAlike 4.0 International License
Rotor – 32.5 mm –fit tester	STL and STEP	Creative Commons Attribution-ShareAlike 4.0 International License
Rotor – 36.6 mm	STL and STEP	Creative Commons Attribution-ShareAlike 4.0 International License
Rotor – PCR strip – 36.6 mm	STL and STEP	Creative Commons Attribution-ShareAlike 4.0 International License
Rotor 36.6 mm – fit tester	STL and STEP	Creative Commons Attribution-ShareAlike 4.0 International License
0.5 ml tube adapter	STL and STEP	Creative Commons Attribution-ShareAlike 4.0 International License
0.2 ml tube adapter	STL and STEP	Creative Commons Attribution-ShareAlike 4.0 International License

**Table:** 

**Design file**	**Description**
Centrifuge body	The main body of the centrifuge, other components are attached to this.
Arrows	Optional – arrows showing the direction of rotation.
Bottom plate	Covers the bottom of the centrifuge.
Rotor – 32.5 mm	Centrifuge rotor for 7 bladed fans with 32.5 mm hub diameter.
Rotor – PCR strip – 32.5 mm	Centrifuge rotor of PCR strips for 7 bladed fans with 32.5 mm hub diameter.
Rotor – 32.5 mm -fit tester	The bottom part of the rotor to test the fit and tightness with the fan hub.
Rotor – 36.6 mm	Centrifuge rotor for 7 bladed fans with 36.6 mm hub diameter.
Rotor – PCR strip – 36.6 mm	Centrifuge rotor of PCR strips for 7 bladed fans with 32.5 mm hub diameter.
Rotor 36.6 mm – fit tester	The bottom part of the rotor to test the fit and tightness with the fan hub.
0.5 ml tube adapter	Adapter for 0.5 ml (500 µl) tubes.
0.2 ml tube adapter	Adapter for 0.2 ml (200 µl) tubes.

***Bill of materials***

## 4 Bill of materials summary

**Table:** 

Designator	Component	Number	Total cost currency	Source of materials
Lid	0.5 l transparent round food container (Curver)	1	2.5 €	https://www.amazon.de/-/en/Curver-Storage-Container-Plastic-Transparent/dp/B06XDP1F7N
PC fan	80 mm, 12 V computer fan with 7 blades	1	4 €	https://www.amazon.de/-/en/AKYGA-AW-8A-BK-Case-Molex-Black/dp/B07TYLRD8S
Lid switch	Hinge lever micro switch 6*9 mm	1	0.2 €	https://www.amazon.de/-/en/Youmile-Switch-Momentary-Button-Arduino/dp/B07YDFH7H3
Main switch	Rocker switch 19*12.5 mm (0.75″*0.5″)	1	0.5 €	https://www.amazon.com/ZUPAYIPA-Solder-Rocker-Switch-Toggle/dp/B01N2U8PK0
Power supply	12 V, 1 A DC power supply, 5.5 mm * 2.1 mm connector	1	9 €	https://www.amazon.de/ppadapter-143-PremiumCord-Universal-Power-Supply/dp/B08439168F
DC socket	5.5 mm * 2.1 mm DC socket	1	1.3 €	https://www.amazon.de/sourcing-pieces-socket-electrical-accessories/dp/B077GKF4FM
Bottom plate screws	M3 * 10–12 mm metric screws	4	0.12 €	https://www.amazon.de/-/en/Countersunk-Hexagonal-Stainless-Wrenches-Furniture/dp/B0915BX7F7
PLA filament	PLA filament for 3D printing	∼ 100 g	2 €	https://www.filanora.eu/filanora-filacorn-pla-bio-hi-filament-175mm-antracit

**Fig. 2:**
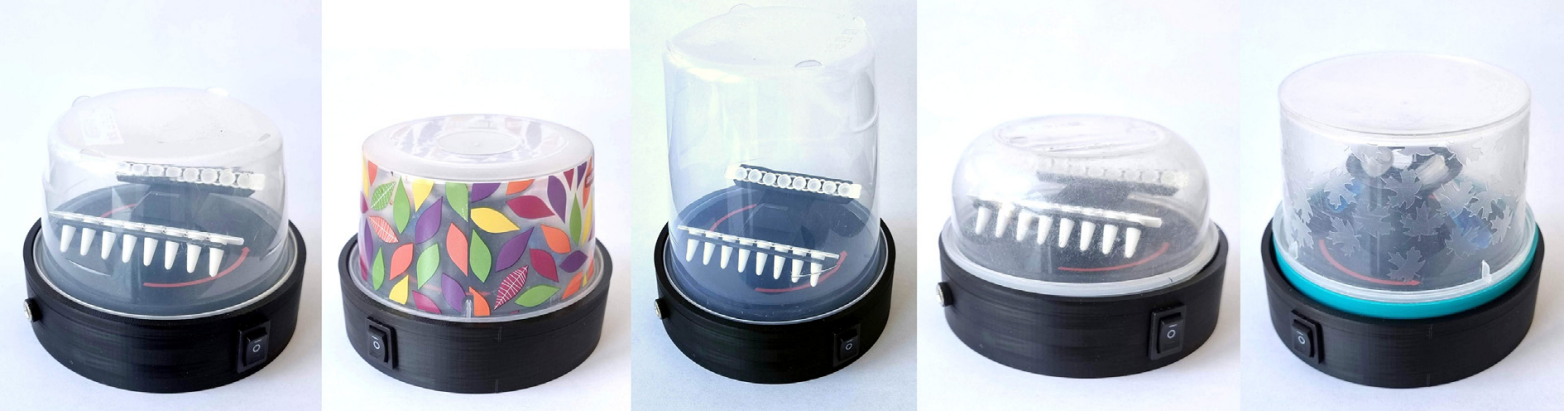
Lid options. The order is the same as listed in the above tables. From left: 0.5 l round (Curver), Deco Chef (Curver), 1 l round (Curver), Cook&Lock (Bager), Ruccola Twist (BranQ).

A transparent food container is used as the lid of the centrifuge. Its diameter is 116 mm which is common for round containers with a volume of approximately 0.5 L. Apart from the one model listed above, the ones in the following table are tested to fit (Table 1 and Fig. 2). If one cannot source a lid matching this dimension, the stl file of an alternative centrifuge body is also uploaded, which allows using containers with 105 mm of minimum and 127 mm of maximum diameter.

**Table 1:** Alternative plastic containers were tested to fit. * not suitable for the PCR strip rotor, **^†^** part of the twist top of the container was cut and glued to the container to widen it and operate the lid switch more reliably.

**Description**	**Cost**	**Source of materials**
Deco Chef round food container set (Curver)*	4 €	https://www.amazon.de/-/en/Curver-Storage-Tins-Storage-Container/dp/B014IX7N5Y

1 l round food container (Curver)	2.8 €	https://www.amazon.de/-/en/00564-139-01-CURVER-Food-storage-container/dp/B010V5YMY8/

Cook&Lock round container 400 ml (Bager)	2.4 €	http://www.bagerplastik.com/en/product-groups/kitchen/storage/cook-lock-round-storage-container-400-ml

Ruccola Twist set (BranQ)*^†^	1.5 €	https://www.amazon.de/-/en/BranQ-essential-Rukkola-BPA-Free-Diameter/dp/B08LQMFQVV/

Important safety notice: never use a glass container as the centrifuge lid!

Of all the materials listed above, the most common versions were chosen, to ensure that most of the components can be bought from small electronics and computer shops. Due to their common nature, all the materials are affordable, but the costs can be decreased further by upcycling used parts: 80 mm fans can be found in computer cases, this type of rocker switch is common in all types of electronic equipment, micro switches are often used in computer mice. The chosen DC connector size is the most widely used type on the market, other types of connectors can be used if available or by soldering the power supply cables directly, the connectors might be skipped altogether.

Wires are not listed, because the cables provided with computer fans can be cut shorter and used for the whole circuitry.

Tools and materials necessary for assembly:

• FDM 3D printer: an Artillery Genius was used to print the prototypes of the manuscript, but any entry-level 3D printer would suffice if meets the following criteria: build plate size at least 13*13 cm, nozzle temperature: 200 °C
• Soldering iron
• Screwdrivers
• Electrical tape or heat shrink tubes
• Glue: E6000 or cyanoacrylate

**Fig. 3:**
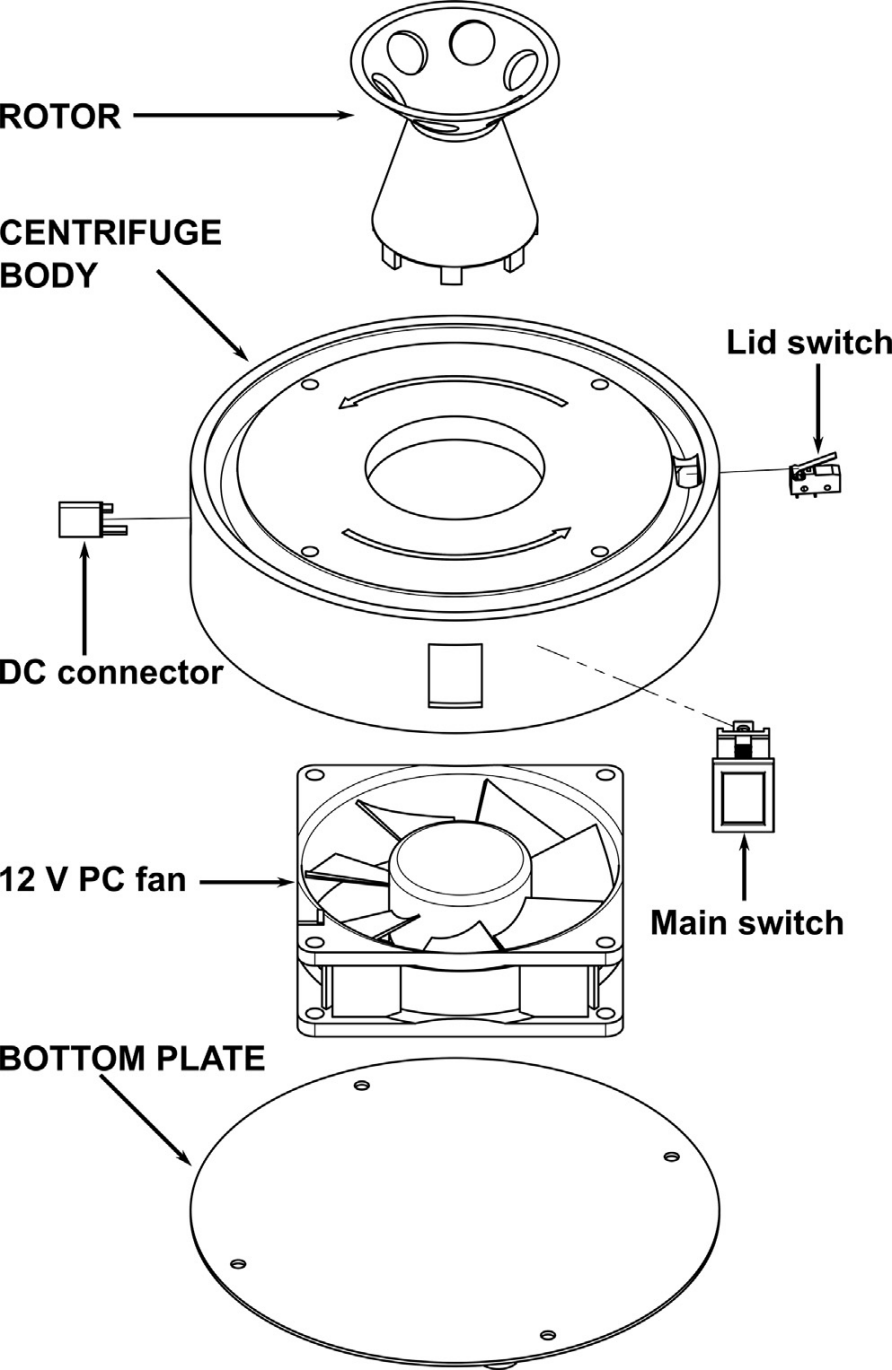
Exploded view of the centrifuge. The 3D printed parts are marked with all caps labels. Models of the fan, main switch and lid switch were downloaded from the McMaster-Carr catalogue. The lid switch is rotated for better visibility. Lid, screws and wires are not displayed.

**Fig. 4:**
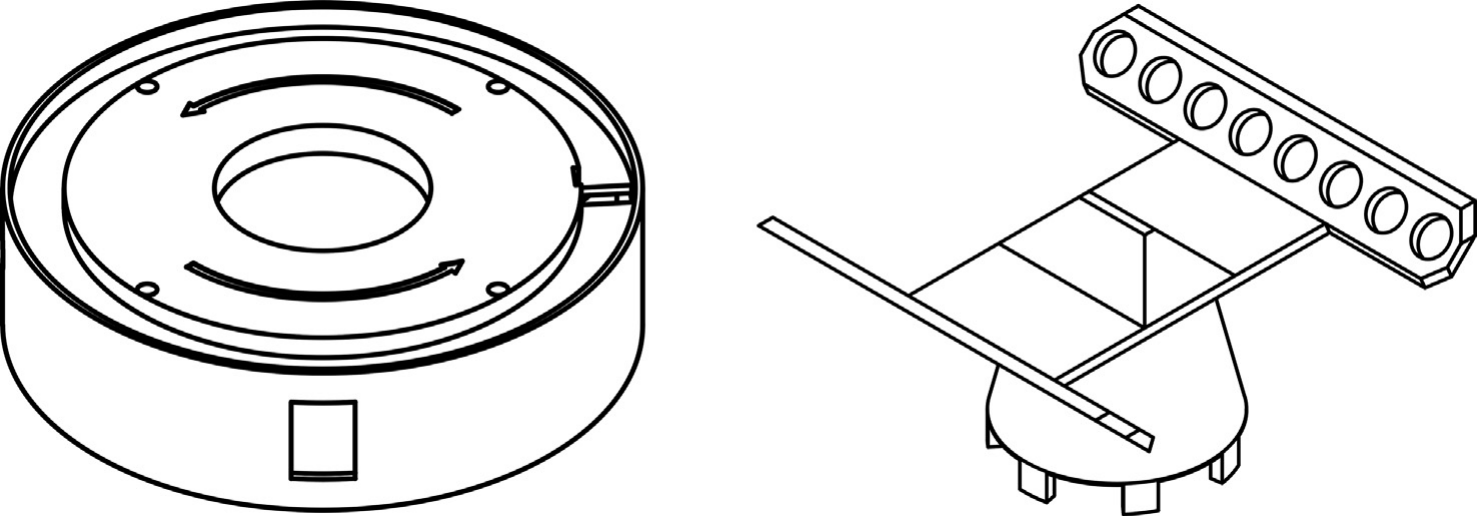
Alternative components: Centrifuge body with broader lid compatibility (left). The widened groove and rotated lid switch make it possible to accommodate lids with 105 mm of minimum and 127 mm of maximum diameter. Rotor for PCR strip tubes (right).

Software used:

• All models were designed with Fusion 360 (Autodesk), which can be used for free with an Educational or Hobbyist license.
• Images were prepared with Fusion 360 and Inkscape (inkscape.org). The latter is free and open source.
• STL models were sliced for 3D printing with the free Cura slicer (Ultimaker) (See Figs. 3 and 4).

## 5 Build instructions

### 5.1 Printing parameters

**Fig. 5:**
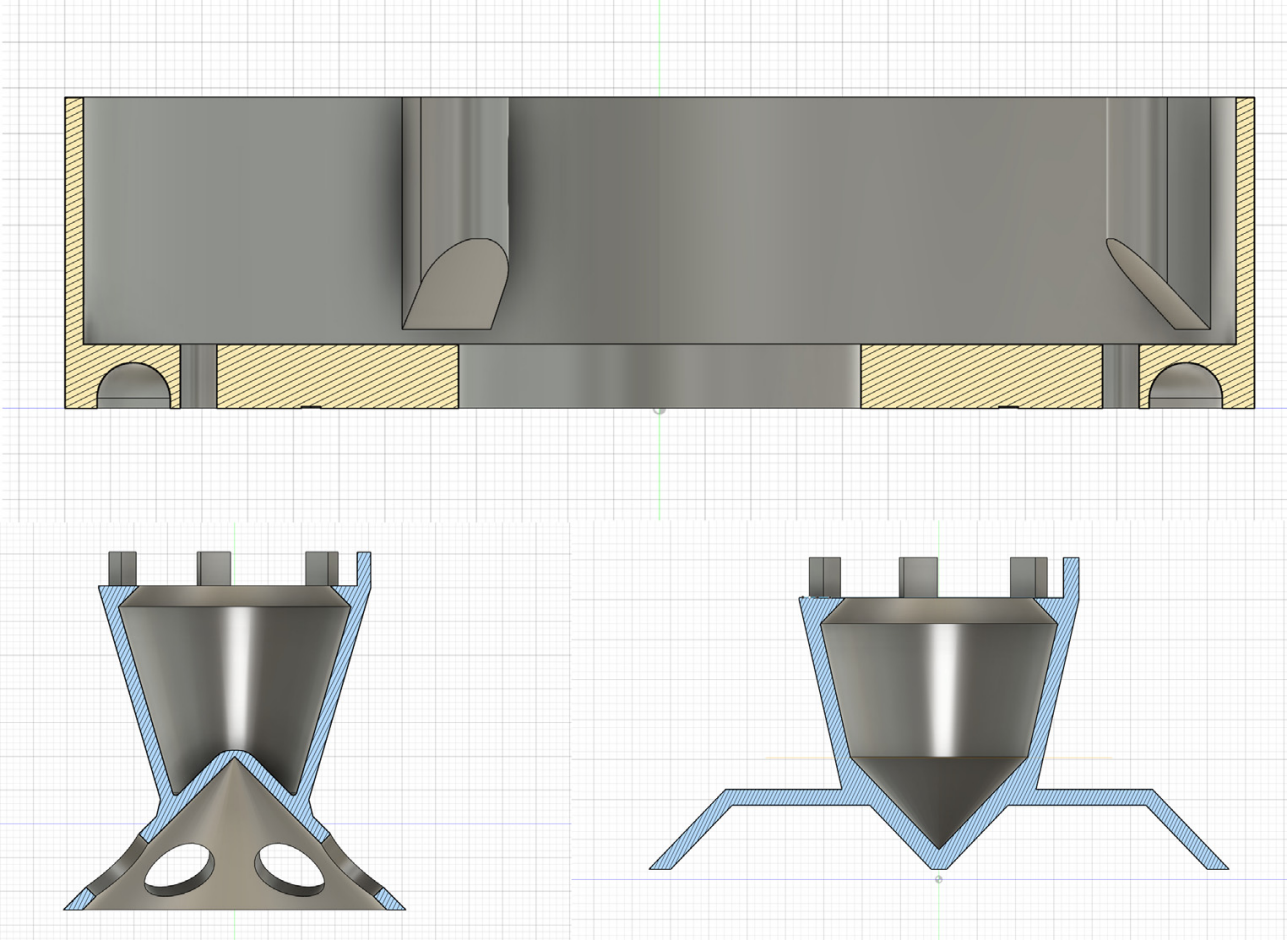
Cross-section view of the centrifuge body (top), rotor (bottom, left) and PCR strip rotor (bottom, right). Shown as the recommended printing orientation.

All models are carefully optimized for supportless printing. All of them should be oriented upside down (Fig. 5). Should print at temperatures recommended by the manufacturer (for PLA usually 200 to 220 °C nozzle, 60 °C bed). Nozzle diameter: 0.4 mm, line width 0.6 mm, 3 wall perimeters.

#### 5.1.1 Printing the centrifuge rotor

**Fig. 6:**
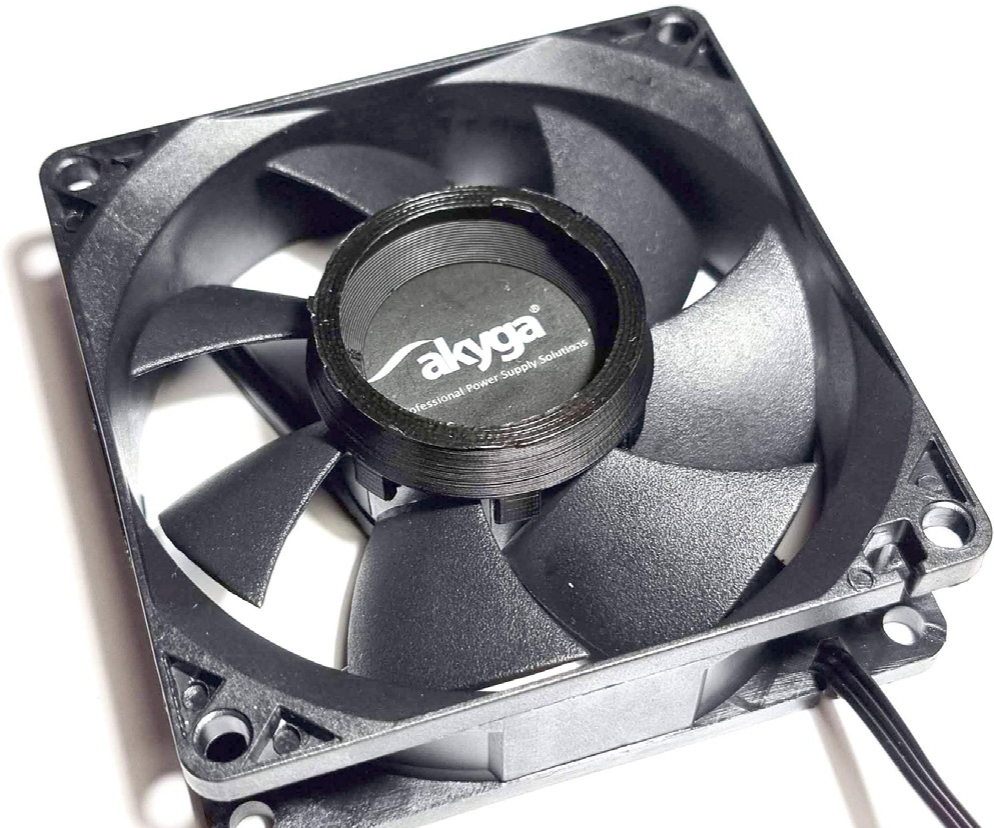
Testing the fit of the rotor on the fan hub with the Rotor – fitting test tool.

Prior printing the full-sized rotor, it is advised to print the Rotor –fit tester tool first and try it on the fan hub (Fig. 6). Measure the fan hub diameter (the two common sizes are 32.5 mm and 36.6 mm) and choose the rotor file accordingly. It should fit snugly. Small adjustments can be performed with the slicer software: if it is too tight use a negative “Horizontal Expansion” in Cura slicer, if it is too loose apply a positive “Horizontal Expansion” value.

To achieve the most balanced weight distribution, the rotor was printed with 100 % infill and random z seams, even if it is not the most pleasing aesthetically.

#### 5.1.2 Printing the centrifuge body

The body of the centrifuge was printed with 10% infill.

**Fig. 7:**
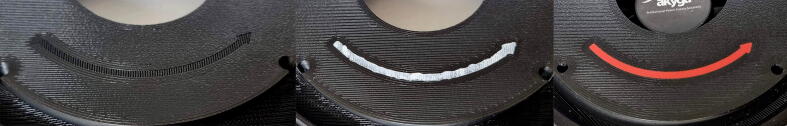
Spin direction arrows. Left: centrifuge body was printed without the inlay. Middle: arrow was highlighted with acrylic paint. Right: Centrifuge body printed with red inlay. (For interpretation of the references to colour in this figure legend, the reader is referred to the web version of this article.)

Spin direction arrows are part of the centrifuge body design. If the centrifuge body is printed as a single object, the arrows are still visible (Fig. 7 **left**). These arrows can be made more prominent by painting with simple acrylic paint (Fig. 7 **middle**).

**Fig. 8:**
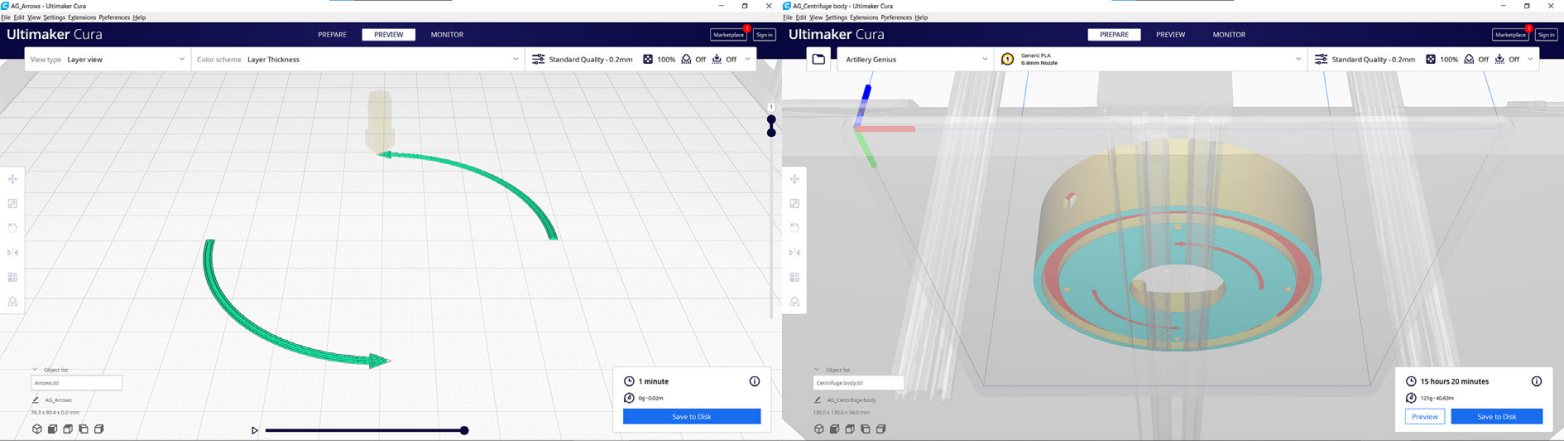
The orientation of the optional spin direction marker arrows and the centrifuge body in Cura slicer.

*Optional:* spin direction arrows printed with different colour filament (Fig. 7 **right**): Slice the Arrows.stl and Centrifuge body.stl files separately, centered on the build plate with 0.2 mm layer height, 0.3 mm z hop, without brim, skirt or raft (Fig. 8). First print the Arrows, when finished change the filament quickly and start printing the Centrifuge body immediately. This way, it is possible to print colourful first layers with a single extruder 3D printer.

### 5.2 Assembling the centrifuge

**Fig. 9:**
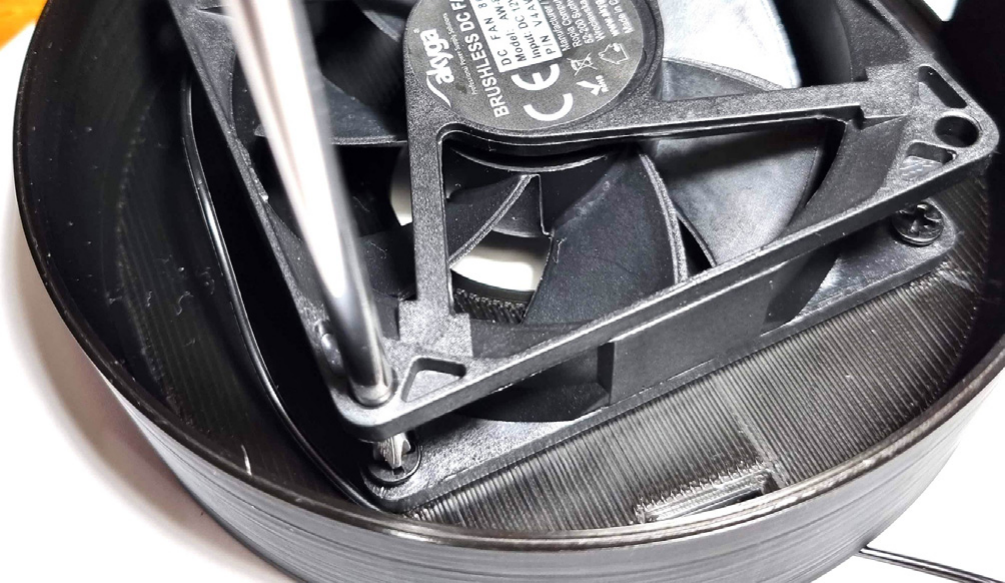
Attaching the fan to the centrifuge body.
• Connect and solder the fan, main switch, lid switch and DC connector according to the circuit diagram (Fig. 10 **left**). Computer fans are usually provided with 30 cm long cables, which can be shortened and the excess can be used to connect the components.

• Fasten the fan to the centrifuge body with the screws which are provided with the fan. (Fig. 9**.**)

**Fig. 10:**
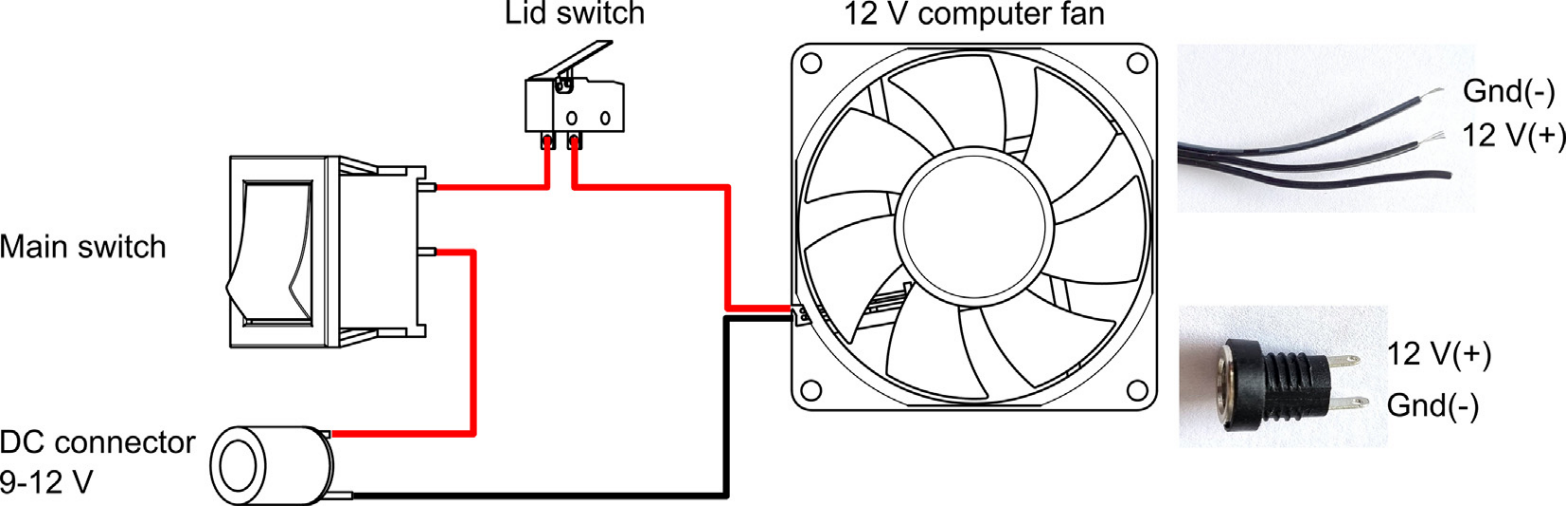
Schematic circuit diagram. Top, right: the three wires of the fan. The ground wire is black with a grey label (barely visible), and the 12 V wire is the middle, black one. Bottom, right: DC jack connector.
• PC fans usually have three wires: Ground, 12 V and speed sensor (Fig. 10 **top, right**). The ground, which is always the first wire (colour: black or black with white or grey label) should be connected to the negative terminal. The 12 V wire is always the middle wire (colour: red or black) that should be connected to the positive terminal. The third wire is irrelevant in our use case. Most fans are protected against reverse polarity, so if the polarity was switched, the fan will not start, but will not suffer irreversible damage. The correct polarity can be checked easily with a 9 V battery.
• Most of the power supplies use a center positive pin at the connector, The shorter terminal of the power connector jack will be the positive terminal and the longer connector will be the negative terminal (Fig. 10 **bottom, right**)
• Attach the rotor to the fan hub by simply pressing on. It should fit snugly. In previous iterations, the fan blades were removed, but it significantly increased the wobbling and resonance of the rotor.
• Place the lid on and start the centrifuge with the unloaded rotor.
• If no resonance can be observed and the rotor runs smoothly (the most likely cause of resonance is, if the rotor is not snapped into its place properly), the rotor can be removed and fixed in the final position with glue (e.g. E6000 or cyanoacrylate).
• Attach the bottom plate with M3 screws.
• Optional: rubber pads could be glued to the rubber feet of the bottom plate.
• If one would need both rotors, it is advised to assemble two complete centrifuge units with glued rotors, rather than switching the rotors.

## 6 Operation instructions

### 6.1 Use the centrifuge only on even, hard surfaces

1. Turn on the main switch.
2. Load the rotor with the tubes. Always properly balance the rotor, by loading it symmetrically! If centrifuging only one or five tubes, use a counterbalance tube filled with water of the same weight as the opposing tube.
3. Place on the lid and press it down slightly to activate the lid switch.
4. To stop the centrifuge, slightly elevate the lid to disengage the lid switch.
5. Always remove the lid until the rotor stops completely.
6. When not in use, keep the main switch turned off.

## 7 Validation and characterization

### 7.1 Testing operation and reliability

The nominal speed of the fan used is 2000 RPM. The real speed of the assembled centrifuge was measured using the super slow motion video mode of a Samsung S21 smartphone: it captures footage at 960 fps for 0.5 s, which made it possible to count the number of revolutions. When the rotor was loaded with two empty adapters (to make counting easier), the measured speed was 2160 RPM, under the load of two 1.5 ml tubes containing 1000 µ liquid, the maximum decreased only to 2100 RPM.

Relative Centrifugal Force (RCF) values were calculated with the following formula: RCF = RPM^2^ × 1.118 × 10^−5^ × *r*, where RPM is the number of rotations per minute, r is the maximum rotational radius in cm. The maximum rotational radius of the six tube rotor is 4.4 cm, using 1.5 ml tubes. The rotational radius of the innermost tubes of the PCR strip is also 4.4 cm. The maximum RCF is 230 G.

While the intended use case of this instrument involves short spinning times (0.5–1 min), it was stress tested by running for 30 min with the load of two 1.5 ml tubes containing 1000 µl liquid. This test was repeated three times without any failures. The room temperature was 22.3 °C, the temperature of the samples was 24.8 °C, and the hub of the fan warmed up to 28.7 °C (measured with a non-contact thermometer (Lidl)). Probably this minimal increase in temperature is tolerable for both the samples and the motor.

The first iteration of this centrifuge (a slightly different, two-part rotor design) is used daily in our forensic genetics lab for about 1.5 years without any problems or failure.

### 7.2 Comparison to commercial counterparts

**Fig. 11:**
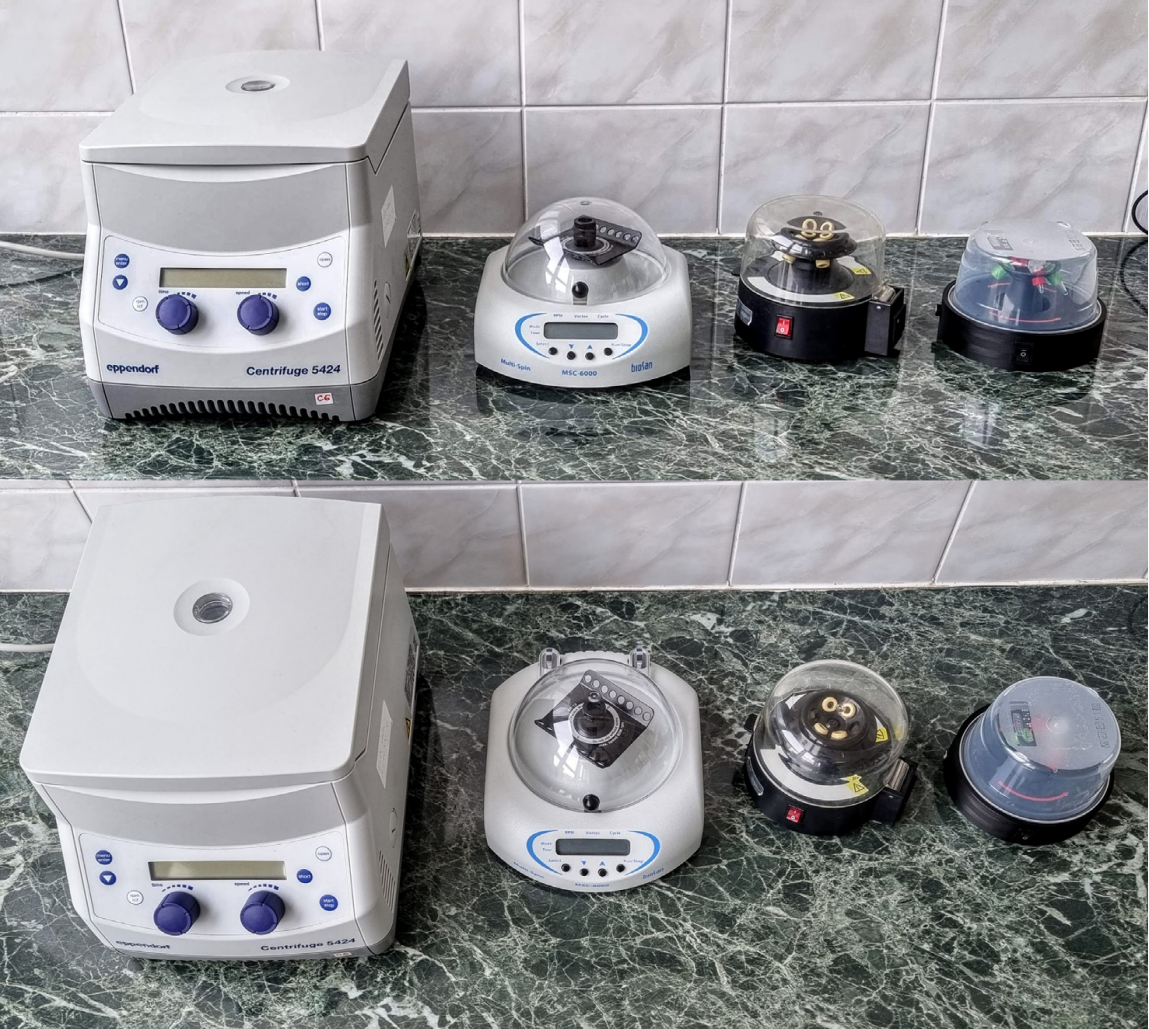
Comparison of dimensions and required benchtop footprint of four centrifuges. From left to the right: Eppendorf Centrifuge 5424, BioSan MSC-6000, Kisker biotech and the DIY open lab spinner.

To test the performance of this DIY centrifuge, it was compared to three commercial small benchtop centrifuges: the Eppendorf Centrifuge 5424, the BioSan MSC-6000 and the Kisker biotech (Fig. 11). The former devices are a lot more capable than the DIY instrument, but they cost several times more (the current Eppendorf 5425 centrifuge costs approximately 3500 Euros, while the BioSan is around 500 Euros) (Table 2).

**Table 2:** Features of the compared centrifuges. *The Eppendorf 5424 is discontinued, the price of the current 5425 model is displayed. **The Kisker biotech centrifuge was bought several years ago, the price is unknown.

Manufacturer	Eppendorf	BioSan	Kisker biotech	DIY
Model	Centrifuge 5424	MSC-6000		
Max. load capacity	24*1.5 ml tubes	12*1.5 ml tube 2 PCR strips	6*1.5 ml tubes	6*1.5 ml tube 2 PCR strips
DimensionsW*L*H (cm)	24*31*22	18.5*24*12	13*15*12	13*13*10
Max. radius (cm) (with1.5 ml tubes)	7.72	5.2	4.4	4.4
Price (EUR)	3500*	500	n.a.**	20

**Fig. 12:**
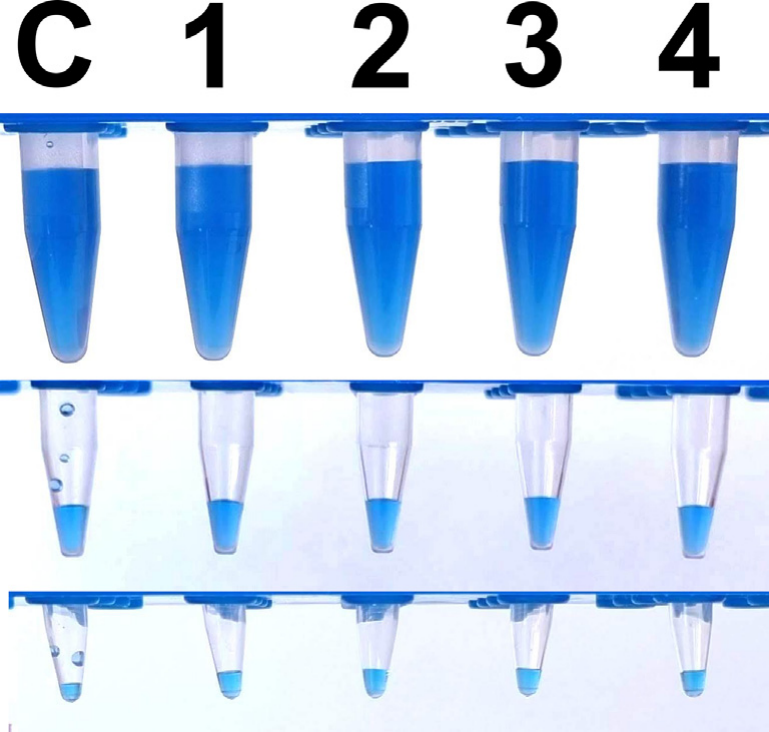
The DIY mini centrifuge showed the same efficacy as the commercial alternatives. Glycerol (20%) – water (80%) – Indigo carmine – mixture was centrifuged for 30 s. C: vortexed sample 1: Eppendorf, 2: BioSan, 3: Kisker biotech, 4: DIY centrifuge. From top to bottom: 1.5 ml tubes – 1000 µl mixture; 0.5 ml tubes – 50 µl mixture; 0.2 ml tubes – 20 µl mixture.

To test the efficacy, a solution of glycerol (20%) – water (80%) – Indigo carmine was created to simulate the consistency of enzyme-containing master mixes. The mixture was vortexed in three different types of tubes (1 ml in 1.5 ml tubes (Sarstedt), 50 µl in 0.5 ml tubes (Thermo Scientific), 20 µl in 0.2 ml tubes (Sarstedt)), then were centrifuged for 30 s. To get comparable results, all centrifuges were set to 230 g (Eppendorf: 1632 RPM, Biosan: 2000 RPM, DIY: 2160 RPM). The Kisker biotech centrifuge lacks speed settings its nominal RPM is 6400 (RCF: 2019 g). All four centrifuges were able to remove the droplets and the liquid film from the wall and cap of the tubes (Fig. 12).

To compare noise levels, all the centrifuges were run with different loads (Table 3) for 30 s. The noise levels were measured with a Samsung S21 phone (Samsung) using the Sound Meter app (https://play.google.com/store/apps/details?id=com.binghuo.soundmeter). The microphone of the phone was held at 50 cm from the front panel of the measured centrifuge. After the centrifuges reached the set speed averaged noise level was measured for 10 s, then averaged by the app (as an average it always rounded the result and gave back an integer).

**Table 3:** Noise levels were measured with different loads.

Model	RPM	Noise level (dB)					
		Empty	20 µl/0.2 ml tubes	50 µl/0.5 ml tubes	1 ml/1.5 ml tubes	PCR strip	Average
Eppendorf	1632	65	64	64	66	na	64.75
Biosan	2000	54	58	58	61	58	57.8
Kisker Biotech	6400	57	57	58	59	na	57.75
DIY	2160	44	45	44	45	45	44.6

The values in Table 3 are meant to compare to each other, and not taken as absolute values. The DIY centrifuge was the quietest among the four instruments. The Eppendorf centrifuge could be almost completely silent, because of the low speed, but a loud cooling fan turned on shortly after the centrifugation started (it is not a refrigerated centrifuge). The DIY centrifuge was also measured in a quiet room and it was not louder than background noise (38 dB).

### 7.3 Safety notes

While this centrifuge spins relatively slow, all the necessary precautions must be taken: always balance the rotor properly. Only use it on solid, stable surfaces. Never use a glass container as a lid. If the device is damaged, do not use it.

After the lid switch is opened it takes 4.2 s (an average of 10 measurements) for the empty rotor to stop. When spinning two 1.5 ml tubes containing 1 ml liquid each, the time stop extends to 5.8 s (average of 10 measurements. It is absolutely advised to wait until the rotor stops completely, but due to the low weight of the rotor (16 g), it has minimal rotational inertia when the motor is not driven.

## Declaration of Competing Interest

The authors declare that they have no known competing financial interests or personal relationships that could have appeared to influence the work reported in this paper.
